# Is titanium alloy Ti‐6Al‐4 V cytotoxic to gingival fibroblasts—A systematic review

**DOI:** 10.1002/cre2.444

**Published:** 2021-05-21

**Authors:** Jonathan Willis, Siwei Li, St John Crean, Fadi N. Barrak

**Affiliations:** ^1^ School of Dentistry University of Central Lancashire Preston UK; ^2^ Department of Materials Royal School of Mines, Imperial College London London UK

**Keywords:** cytotoxicity, implant, peri‐implantitis, Ti‐6Al‐4 V alloy

## Abstract

**Objectives:**

Grade V titanium alloy (Ti‐6Al‐4 V) is a well‐recognized metallic biomaterial for medical implants. There has been some controversy regarding the use of this alloy in medical devices in relation to the toxicity of vanadium. In Dentistry, Ti‐6Al‐4 V remains prevalent. This systematic review aims to evaluate the effects of Ti‐6Al‐4 V on cells relevant to oral environments such as gingival fibroblasts.

**Materials and methods:**

A literature search was undertaken for relevant English language publications in the following databases: Dental and Oral Science, Medline and Web of Science. The electronic search was supplemented with a search of references.

**Results:**

After application of inclusion and exclusion criteria. A total of eight papers are included in this review. These papers were all in vitro studies and were categorized into whole implant, discs, or implant particles based on the type of test materials used in the studies.

**Conclusion:**

Based on the analyses of the eight included studies in this review, if Ti‐6Al‐4 V as a material is unchallenged, i.e., as a whole implant in pH neutral environments, there appears to be little effect on fibroblasts. If Ti‐6Al‐4 V is challenged through corrosion or wear (particle release), the subsequent release of vanadium and aluminium particles has an increased cytotoxic effect in vitro in comparison to commercially pure titanium, hence concerns should be raised in the clinical setting.

## 1 INTRODUCTION

Dental implants can be used to stabilize dentures, replace missing teeth through bridges or crowns and act as anchorage for orthodontic treatment. Denture use is decreasing as the population demands better options to replace missing teeth (Jivraj & Chee, 2006). The ideal dental implant material was described by Osman and Swain as possessing a number of features including biocompatibility, adequate toughness, sufficient strength, as well as corrosion, wear and fracture resistance (Osman & Swain, 2015). There is currently no perfect material that meets these criteria. To achieve this, implants are evolving and are currently classified as metal, ceramic or polymers (Medvedev et al., 2016). This systematic review will focus on metal implants.

The majority of metallic biomedical implants can be divided into commercially pure titanium (cpTi) grades I‐IV and alloys, grade V. The strength of commercially pure titanium grade IV (the strongest grade) is 550 MPa. To increase this strength a titanium alloy can be used, e.g. a combination of titanium, aluminium and vanadium results in a strength of 930 MPa (Zhang & Chen, 2019). The most commonly used alloy in implant dentistry is grade V, an alloy of titanium, aluminium and vanadium (Ti‐6Al‐4 V). There is also Ti‐Zr/Roxolid^©^, an alloy of titanium and zirconia. The advantages of a stronger dental implant are that narrower or shorter implants can be placed without increasing the risk of implant fracture during function, overcoming the need for complicated vertical bone augmentation (Esposito et al., 2006) in certain situations and offering a reliable option in more challenging locations (al‐Nawas et al., 2012; Hallman, 2001) such as narrow ridge defects.

The survival rate for dental implants has reached in excess of 90% after 2–7 years in function with high scores of patient satisfaction at 88% (Al‐Hamdan, 2018; Bazrafshan & Darby, 2014). In Implant Dentistry, the most common late presentation complication is plaque induced peri‐implantitis (Saulacic & Schaller, 2019). Peri‐implantitis is a plaque‐associated pathological condition occurring in tissues around dental implants (Berglundh et al., 2018). It is characterized by inflammation in the peri‐implant mucosa and subsequent progressive loss of surrounding supporting bone in which the implant is anchored. Derks and Tomasi reported in their systematic review with meta‐analysis on the prevalence of peri‐implantitis to be 1%–47% with selected implant systems (Derks & Tomasi, n.d.). Metal particles have been found in dental peri‐implant mucosa with and without peri‐implantitis, though particle concentration was higher in sites with peri‐implantitis (Olmedo et al., 2013). Particles were found both inside and outside of epithelial cells and macrophages and in bone and soft tissue of affected sites (Fretwurst et al., 2016).

Particles are released from dental implants through shear forces at insertion, wear from the abutment‐implant interface and implant maintenance through ultrasonic scaling and implantoplasty (Delgado‐Ruiz & Romanos, n.d.). Corrosion cycles initiated by exposure to saliva, bacteria and/or chemicals that can affect the titanium oxide layer as well as other factors such as micro‐gap (e.g. at implant‐abutment connection) and fluorides can result in the release of particles (Delgado‐Ruiz & Romanos, n.d.; Suarez‐Lopez Del Amo et al., 2018). Knowledge of how dental implants interact with their environment is changing. Metallic particles smaller than 10 microns in diameter can be internalized by cells (Dalal et al., 2012). The possible effects of this include potential cytotoxicity, chromosomal damage and increased oxidative stress. The effects of vanadium compounds have been described as being carcinogenic, immunotoxic and neurotoxic (Zwolak, 2014). Taus et al. also described that aluminium induces the expression of inflammatory and pro‐apoptotic genes (Taus et al., 2013). Challa et al. have described how the orthopedic community have moved away from the use of vanadium containing alloys in orthopedic implants due to the concern of released vanadium and since 1985, non‐vanadium containing alloys have been used more widely (Challa et al., 2013). The dental community continues to use this alloy as an anchorage aiding orthodontics in teenage subjects, dental implant body and as an abutment material. As implant connections are mobile between abutment (e.g., grade V) and implant body (e.g., grade IV), there is a significant possibility of differential wear, releasing particles.

Joint replacement procedures and dental implants have excellent rates of success but when failure occurs it is an expensive modality to repair or replace. It is estimated that a hip replacement procedure costs the NHS £5620 and a knee replacement £5350 (The Guardian, 2014). The average cost of a dental implant is reported as £2334 (Dentistry.co.uk, 2017). Failed dental implants can be replaced if there is sufficient bone, however bone grafting procedures may be needed incurring additional costs (Zhou et al., 2016). As the medical technology industry strives for stronger and longer lasting implant materials, one should question whether the current materials used may possibly exert long term negative effects? It is only recently that research in the dental field has looked at wear particles from dental implants and whether these are found in oral soft and hard tissue and the effect these could have (Fretwurst et al., 2016; Mombelli et al., 2018; Suarez‐Lopez Del Amo et al., 2018). As there is evidence that shows that particle and ion release will occur from dental implants, it is therefore important for clinicians to understand what the local tissue response and consequence could be. There has not been a previous systematic review on the effect of Ti‐6Al‐4 V on fibroblasts, a clinically relevant cell that plays an important role in the integration of dental implants. If Ti‐6Al‐4 V titanium alloy has a cytotoxic effect on fibroblasts, then this could be part of a mechanism of failure. This systematic review aims to assess the current published literature as to whether Ti‐6Al‐4 V has a cytotoxic effect on fibroblasts in vitro.

## 2 MATERIALS AND METHODS

The search strategy was compiled using the PICO framework (Supplementary Table 1): (1) Population: In vitro studies involving fibroblasts and a biomedical implant utilizing Ti‐6Al‐4 V as a component, themes searched were terms regarding titanium alloy, Ti‐6Al‐4 V; (2) Intervention: Implant release of particles and/or ions, themes searched were terms regarding wear and release metal particles and ions; (3) Comparison: Comparative studies involve other implant alloys and/or commercially pure titanium grades I‐IV, themes searched were terms regarding commercially pure titanium and/or other titanium alloys not Ti‐6Al‐4 V, and (4) Outcome: Effects on fibroblasts. The search words and Boolean operators are presented in Supplement Table 1.

### 2.1 Data sources

**FIGURE 1:**
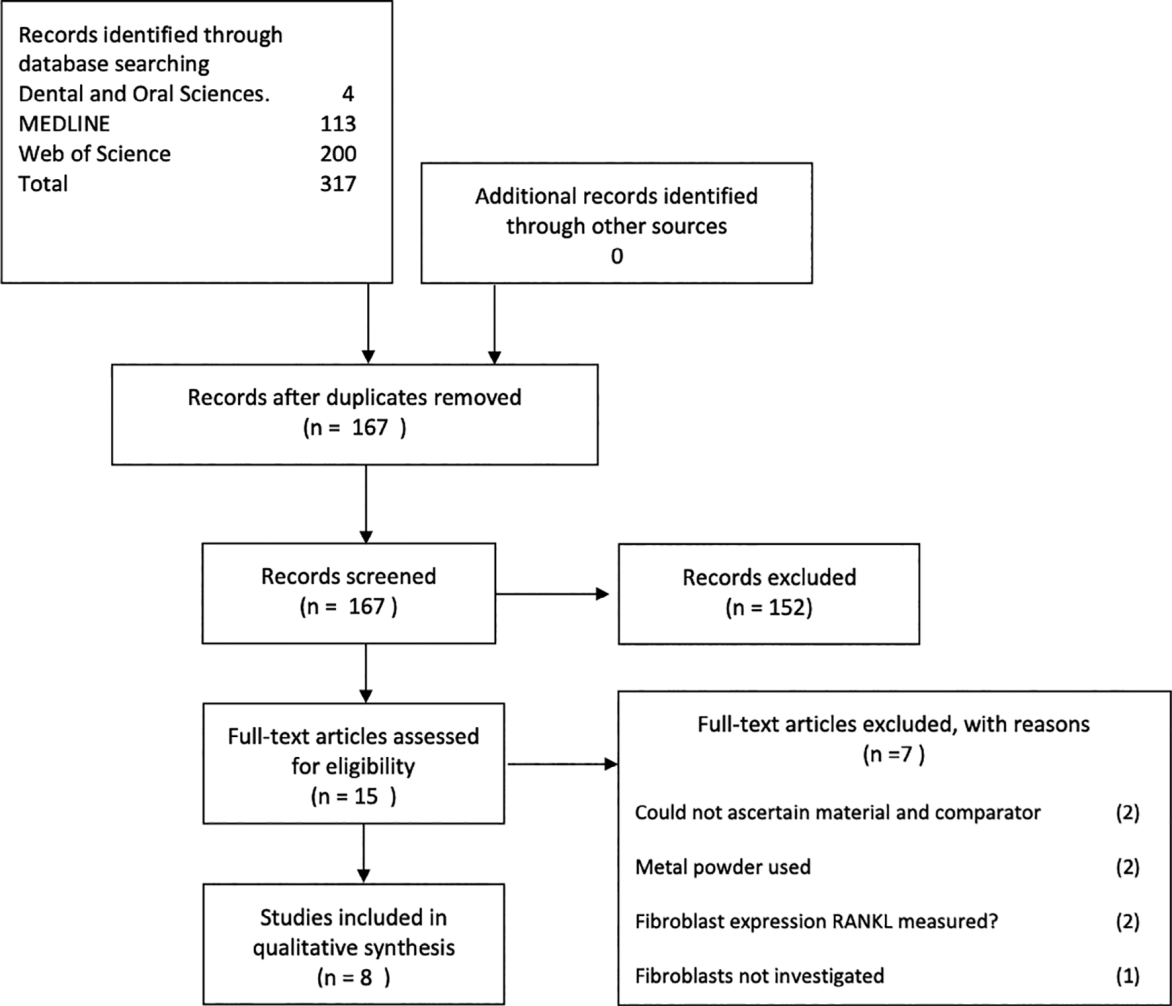
PRISMA workflow

A PRISMA (preferred reporting Items for systematic reviews and meta‐analyses) workflow was used to search relevant publications in the following databases (Figure 1): Dental and Oral Science, Medline and Web of Science. Only in vitro studies were included in this review as human studies are not available due to ethical issues of removing successful biomedical implants nor biopsies of gingivae or adjacent tissue to prosthetic joints for the purpose of assessing wear and the release of particles and no animal studies were included due to the lack of evidence of particle release in limited study durations. Non‐English language publications and articles with no control/ comparator were excluded. A hand search of references was also undertaken. In accordance to Cochrane review guidelines, there is currently no quality and bias assessment method that has been developed for analysis of in vitro studies. Due to the lack of commercial sponsorship, a low risk of bias can be concluded from all studies included in the present review.

### 2.2 Data extraction

A total number of eight articles were included in the current review. Exclusion process and number of excluded articles were presented in the Prisma workflow chart (Figure 1). Publications included in this review were categorized by the form of the Ti‐6Al‐4 V alloy tested, as whole implant, disks or particles. A summary of articles included in this systemic review is presented in Table 1. The outcome to be measured was whether Ti‐6Al‐4 V has an adverse in vitro cellular/biological effect on fibroblasts.

**TABLE 1:** Summary of publications included in this systematic review

	Galeotti et al. (2013)	Malkoc et al. (2012)	Kim et al. (2008)	Markhoff et al. (2017)	Chander et al. (2017)	Mostardi et al. (2010)	Dalal et al. (2012)	Evans (1994)
Test Materials	Stainless Steel Grade IV Ti‐6Al‐4 V	Stainless Steel Ti‐6Al‐4 V	Grade 2 Ti‐6Al‐4 V	Ti‐6Al‐4 V Ti‐6Al‐4 V additive manufactured NiTi NiTi DLC	Grade 1 Ti‐6Al‐4 V	Cp‐Ti Cp‐Ta Ti‐6Al‐4 V Co‐Cr‐A Co‐Cr‐B	Ti‐6Al‐4 V Co‐Cr‐Mo Zr‐oxide Zr‐alloy	Ti Ti‐6Al‐4 V Co‐Cr‐Mo
Size	Whole implant	Whole implant	Discs 5 mmx 3 mm	Disks 10 mm x 2 mm	Disks 8 mm x 5 mm	Particle (Size not reported)	Particle 0.1 to 1 μm	Particle Max 45 μm
Fibroblast origin	Human gingival fibroblasts	Human gingival fibroblasts	Mouse fibroblasts 3 T3	Fibroblasts human skin biopsies, osteoblasts, macrophages	Human gingival fibroblasts	Human synovial fibroblasts	Human fibroblasts (origin not specified)	Fibroblasts ‐ rat skin)
Culture methods	Cell culture in implant eluates	Cell culture in implant eluates	Days 2 + 5 proliferation 16 hrs adhesion	96 Hours	48 and 72 hours	5 days	48 hours	2,4,6 days
Variable	Test material, pH and time	Test material and time	Time	Alloy, cell time	Time different solutions	Particle concentration	Particle concentration	Direct/indirect contact
Analysis	MTT metabolic activity assay	xCELLigence cell survival	MTT Proliferation assay Adhesion	WST‐1 Inflammatory cytokine assay	MTT Proliferation assay	Trypan blue cell count	ATP chemiluminescence Proliferation LDH assay Inflammatory cytokine assay	Cells counted
Findings	Ti‐6Al‐4 V eluates (pH 4) resulted in significant (*p* < 0.01) cytotoxicity	Ti‐6Al‐4 V whole implant is not cytotoxic	Vanadium ion effects proliferation	Ti‐6Al‐4 V increase MMP‐1	Ti‐6Al‐4 V reduced cell viability	Reduced cell number (large patient variation	Decreased proliferation, upregulated inflammatory marker of fibroblasts in a dose dependant manner	Reduced cell number

Whole implant: Two studies examined the effect on fibroblasts from whole implants (Galeotti et al., 2013; Malkoc et al., 2012). Both studies used eluates/dissolution products from whole implants to assess the effect on human gingival fibroblasts (HGFs). MTT (Methylthiazolyldiphenyl‐tetrazolium bromide), a cellular metabolic activity assay (Galeotti et al., 2013), and xCELLigence real time cell analyzer system (Malkoc et al., 2012) were used as means to assess cell viability. Galeotti et al immersed 3 types of grade V miniscrews, Miniscrew Anchorage System® (Micerium, Avegno, Italy), Spider Screw Anchorage System® (HDC, Sarcedo, Italy) and Ortho Screw® (Novaxa, Cinisello Balsamo, Italy) in 0.9% (w/v) NaCl solution at pH 7 and 4 for 1, 14, 21, 28, and 84 days (Galeotti et al., 2013). These 2 conditions were chosen to mimic normal and reduced physiological pH of saliva, which has a normal pH range of 6.2–7.6 and reduced pH as a result of food intake and microbial flora (Baliga et al., 2013; Galeotti et al., 2013). The dissolution products were then diluted in standard culture media (Dulbecco's minimum essential media) to 25% (v/v) in accordance to ISO 10993‐5 standards. Dissolution products, i.e. ionic products of test materials dissolution in culture medium, of all 3 types of implants from just 1 day of immersion in pH 4 solution, as well as those from longer immersion periods, resulted in significant reduction of HGF viability. Dissolution products from implants immersed in pH 7 solution on the other hand appear to be biocompatible under the same culture conditions. In study conducted by Malkoc et al, dissolution products were prepared by immersion of implants including AbsoAnchor® (Dentos, Daegu, South Korea), MTN® (MTN, Istanbul, Turkey), IMTEC Ortho® (3 M Unitek, Oklahoma, US) and VectorTAS® (Ormco, California, US) in DMEM (Dulbecco's modified eagle's culture medium) for 72 hours and, there was no adverse cytotoxic effect on fibroblasts (Malkoc et al., 2012). The pH of DMEM was maintained at 7.2–7.4. Neither study reported the ionic concentration of metallic elements such as aluminium and vanadium in tested dissolution products, their direct effects on gingival fibroblasts therefore cannot be concluded.

Disks: Three studies have utilized Ti‐6Al‐4 V alloy disks as test materials (Chandar et al., 2017; Kim et al., 2008; Markhoff et al., 2017). Kim et al cultured mouse fibroblast cell line 3 T3 directly on mirror polished grade II cpTi and Ti‐6Al‐4 V alloy disks (5 mm diameter and 3 mm thickness) for the analyses of cell attachment and metabolic activity. No difference between cpTi, Ti‐6Al‐4 V and polystyrene tissue culture plastic were reported following 5 days of culture (Kim et al., 2008). The ionic concentrations of metallic elements in the medium following the culture period were not reported. In the study conducted by Chander et al, dissolution products from 7 grade I cpTi and Ti‐6Al‐4 V alloy disks (8 mm diameter and 5 mm thickness) were prepared and primary human gingival fibroblasts were cultured in standard cell culture medium containing 5, 10, 25, 50 or 100 μL of each dissolution product (Chandar et al., 2017). The authors however did not provide details of the preparation methods nor quantification of final Ionic concentration of metallic elements. Nevertheless, Ti‐6Al‐4 V resulted in reduced cell metabolic activity with increased sample dose following 48 hours of culture.

Markhoff et al cultured primary human fibroblasts (breast and skin), osteoblasts and macrophages directly on forged and selective laser melted Ti‐6Al‐4 V alloy disks (10 mm diameter and 2 mm thickness) for the investigation of viability (Live‐Dead staining), metabolic activity (WST‐1 assay) and inflammatory response (qPCR and ELISA assay) (Markhoff et al., 2017). While selective laser melted Ti‐6Al‐4 V alloy did not result in noticeable effects on all cell types, cells cultured on forged Ti‐6Al‐4 V alloys, though not statistically significant, demonstrated reduced metabolic activity as well as upregulated markers such as MMP‐1 (Matrix metalloproteinase‐1), at both gene and protein levels, an indication of increased inflammatory response. It appears manufacturing process of Ti‐6Al‐4 V alloys have an impact on its corrosion resistance. While Markhoff et al conducted the most comprehensive analyses of cellular response, amongst these studies, only Kim et al reported surface characteristics such as roughness and contact angle of tested alloy disks which could affect cellular attachment and subsequent activities. None reported ionic concentrations of metallic elements following the culture period.

Particles: Three further studies examined the biological effect of Ti‐6Al‐4 V particles (Dalal et al., 2012; Evans, 1994; Mostardi et al., 2010). In the study by Dalal et al. human THP‐1 monocyte cell line and fibroblasts (tissue origin unspecified) were cultured in presence of Ti‐6Al‐4 V alloy particles with 1.3 μm in diameter at concentrations of 5, 10, 50, and 100 particles per cell (Dalal et al., 2012). There was no significant increase in lactate dehydrogenase (LDH), an enzyme released into extracellular space when cellular plasma membrane is damaged, nor significant reduction in ATP synthesis, indicating there was no adverse effect from Ti‐6Al‐4 V alloy particles in terms of viability to both cell types. However, Dalal et al. reported that Ti‐6Al‐4 V alloy particles caused significantly decreased cell number/proliferation in both monocytes and fibroblasts in a dose‐dependent manner in comparison to control at 50 and 100 particles per cell. No significant change in cell number/proliferation was observed at 5 and 10 particles per cell. Cell proliferation was assessed using thymidine incorporation assay. One possibility is that the incorporation of thymidine into new strands of chromosomal DNA during mitotic cell division was affected by metallic ions from Ti‐6Al‐4 V. However, it should be noted that the radiochemical thymidine itself has been reported to induce cell‐cycle arrest and apoptosis in addition to DNA damage (Hu et al., 2002). Dalal et al. also reported upregulation of the production of inflammatory markers such as IL‐1β, IL‐6 and TNF‐α by both the fibroblast and monocyte cell lines at all tested concentrations.

Mostardi et al isolated primary human synovial fibroblasts from 4 donors undergoing total knee arthroplasty (mean age, 64 years, age range 48–73 years, 3 female and 1 male) and cultured in the presence of 4 and 40 mg/mL cpTi (grade unspecified) or Ti‐6Al‐4 V alloy particles (Mostardi et al., 2010). Both cpTi and Ti‐6Al‐4 V alloy particles reduced cell number in a dose‐dependent manner though no statistical difference between the two. Mostardi et al also revealed noticeable patient variation, suggesting resilience to metallic particle/ions could be dependent on patient age, sex and/or other pre‐existing health conditions. Although the authors suggested the particles were internalized by the fibroblasts via lysosomal engorgement and activity, the particle size was not reported. Evans in another study investigated the effect of cpTi (grade unspecified, ground particle mean diameter of 14 μm, unground particle mean diameter of 49 μm) and Ti‐6Al‐4 V alloy particles (mean diameter of 8.9 μm) on primary rat skin fibroblasts (0.5 mg of particles per 10^4^ cells) (Evans, 1994). When cells were directly exposed to ground cpTi or Ti‐6Al‐4 V alloy particles, a significant reduction in cell number was observed following 2 days of culture in comparison to no‐particle controls. Unground cpTi particles did not have any adverse effect. Interestingly, when cells were exposed to these metal particles indirectly, i.e. cells were separated from particles using 0.4 μm pore size cell culture inserts, none of these metal particles had an impact on cell number. While the adverse effect could be the result of metallic ion release internally through phagocytosis, it remains unclear what is the mechanism by which direct contact with particles affect cells and it has been suggested that it may be due to damage to the cell membrane (Evans & Clarke‐Smith, 1991).

## 3 DISCUSSION

The purpose of this systematic review was to address the question as to whether grade V titanium alloy Ti‐6Al‐4 V is toxic to cells in the peri‐implant environment. Within this review, of the eight in vitro studies, there was limited homogeneity for direct comparison to take place. In addition, none of these studies quantified the actual ionic concentration the cells experienced. Nevertheless, it can be inferred that if Ti‐6Al‐4 V alloy is not challenged, e.g. in acidic conditions, there is little or negligible adverse effect to fibroblasts (Galeotti et al., 2013; Kim et al., 2008; Malkoc et al., 2012; Markhoff et al., 2017). Ti‐6Al‐4 V biomedical implants as all titanium biomedical implants when in contact with air will form an oxidized layer, which enhances biocompatibility. The Ti‐6Al‐4 V alloy, as a biomedical implant, however is rarely employed as an unchallenged material. Newly published studies have shown that dental implants will wear and release metallic particles (Delgado‐Ruiz & Romanos, n.d.; Suarez‐Lopez Del Amo et al., 2018).

The results from Galeotti et al. and Malkoc et al. suggest that under normal physiological pH, Ti‐6Al‐4 V implants as a whole, appear to be biocompatible (Galeotti et al., 2013; Malkoc et al., 2012). However, food intake and microbial flora can induce a decrease in the physiological pH of saliva (5.3 to 7.8) (Aamdal‐Scheie et al., 1996; Humphrey & Williamson, 2001). The decrease of cellular metabolic activity caused by implant dissolution products, as a result of immersion in acidic solution, was likely due to the increased release of metal ions from the tested implants, as low pH can affect corrosion resistance. It has been reported that a greater number of metal ions were released in solution from orthodontic implants immersed in acid solutions (Ahn et al., 2006; Kao et al., 2007; Staffolani et al., 1999). Neither study however reported the ionic concentration of metallic elements such as aluminium and vanadium in tested dissolution products.

Indeed, when Ti‐6Al‐4 V alloy is challenged either through corrosion (Chandar et al., 2017; Galeotti et al., 2013) and/or wear particle formation (Dalal et al., 2012; Evans, 1994; Mostardi et al., 2010), the cytotoxic effect of Ti‐6Al‐4 V alloy on fibroblast is evident from the studies presented in this current systemic review. Since it is evident Ti‐6Al‐4 V is very well tolerated in bulk form, it is reasonable to propose that the toxic effect from Ti‐6Al‐4 V reported by both Evans and Mostardi et al studies was due to direct cellular uptake of micro and/or sub‐micron metallic particles, which previously has been shown to be toxic in vitro to several types of human cell populations, such as lymphoblastoid and hepatoma cells (Wang, Mao, et al., 2007b; Wang, Sanderson, & Wang, 2007a). Titanium (oxide) wear particles from Orthopedic implants have also been reported to enter bone‐forming cells and stem cells in vitro via endocytosis and cause adverse biological response such as osteolysis (Haleem‐Smith et al., 2012; Okafor et al., 2006; Yao et al., 2017). Although dental implants are relatively small in size and less susceptible to heavy cyclic mechanical loadings, particles are released from dental implants through shear forces at insertion, wear from the abutment‐implant interface, implant maintenance as well as corrosion (Delgado‐Ruiz & Romanos, n.d.; Suarez‐Lopez Del Amo et al., 2018). There is increasing awareness of the toxicity from the release of low level vanadium ions from orthodontic implants (Costa et al., 2019; Hanawa, 2002; Okazaki & Gotoh, 2013), which has been recorded in kidney, liver and lung (de Morais et al., 2009). It has also been shown in vitro that fibroblasts can induce osteoclastogenesis through RANKL (nuclear factor kappa‐Β ligand, a regulator of osteoclast formation, activation and survival) expression when exposed to Ti‐6Al‐4 V particles (Jiang et al., 2013; Sakai et al., 2002). Despite the studies by Mostardi et al., Dalal et al. and Evans demonstrated potential toxic effects by particles form Ti‐6Al‐4 V, authors acknowledge that the weakness of these studies was the lack of quantification of particles and/or ionic concentration. Previous studies have demonstrated that the toxic effect of V and Al on animal cells were dependant on material concentrations (Okazaki, 2001; Okazaki et al., 1998). Reduced metabolic activities in human gingival fibroblasts when exposed to as low as 0.116 ± 0.023 ppm V was previously reported (Barrak et al., 2020). Other studies have reported that particle size, which affects their ability to inflict cell damage, also has an effect on cytotoxicity (Omar Zaki et al., 2015; Park et al., 2011). In this review, only studies by Dalal et al and Evans reported particle size of Ti‐6Al‐4 V (0.1–1 μm and 45 μm respectively) (Dalal et al., 2012; Evans, 1994). Due to different experimental procedures adopted by the two studies, authors could not perform direct comparison and draw conclusions on the effects of Ti‐6Al‐4 V particle size on human fibroblasts.

The Orthopedic community compiled a national joint replacement registry recording interventions, outcomes, survival and surgical technique used (National Joint Registry, 2019). Any patient with a metal on metal hip replacement is advised to have regular blood tests to measure the level of metal ions such as chromium and cobalt in their body (Medicines and Healthcare Products Regulatory Agency, 2017). In Dentistry, there is currently no such registry. Dentists are required to monitor their own success rate and log each implant placed. In a referral setting, e.g. between individual implantologist and dental practitioner, however, the risk of losing the patient following the fitting of the crown has been high. The Orthopedic community are actively researching alternative materials and studies are using Ti‐6Al‐4 V alloy as a reference. Authors would like to raise the awareness in the dental community that Ti‐6Al‐4 V implants, once corroded intra‐orally, will likely release toxic metallic and ionic products such as vanadium into both soft and hard tissues and, there is increasing evidence that such products of corrosion and wear from dental implants may have a negative biological effect. There are pressing needs for further investigations and without collective efforts, the dental community may be missing important knowledge that could help improve the dental implant and patient quality of life. Authors believe improved understanding of the adverse effect from any implant material should therefore come from studies, if possible in vivo, of interactions between local cells and particles rather than the compatibility of the bulk material. To do so, animal and human subjects will be required, however, there remain considerable practical, ethical and moral barriers. In order to gain further insights into the effect of implant wear particles, both local particulate and ionic concentrations should be recorded in future studies. Furthermore, it is crucial that the design of implants should become more focused on avoiding the production of fine wear particles.

## 4 CONCLUSION

As discussed above, based on the analyses of the included studies in this review, the effect of Ti‐6Al‐4 V can be at a cellular level and is dependent on local metallic particle and/or ionic concentration. The studies identified in this review were in vitro models for cytotoxicity analyses. One should note it is difficult to accurately extrapolate these findings into in vivo and/or real clinical use in human subjects, though it is probable that tissue reactions to Ti‐6Al‐4 V in vivo are also due, at least in part, to the same mechanism such as direct contact with Ti‐6Al‐4 V particulate and ionic matters presented in these in vitro studies here.

## CONFLICT OF INTEREST

The authors declare no conflict of interest.

## AUTHOR CONTRIBUTION

JW contributed to conception, design, data acquisition, analyses and interpretation, drafted and critically revised manuscript. SL contributed to conception and design, critically revised and edited manuscript. SC contributed to review of manuscript. FB contributed to conception and design, critically revised manuscript. All authors gave their final approval and agree to be accountable for all aspects of the work.

## Supporting information


**Supplementary Table 1**: Search words and Boolean operators

## Data Availability

Data available from corresponding author upon request.
