# Supplementary material for: Is titanium alloy Ti‐6Al‐4 V cytotoxic to gingival fibroblasts—A systematic review
Source: Clin Exp Dent Res. 2021 May 21;7(6):1037–44. doi: 10.1002/cre2.444 (PMC8638288; doi:10.1002/cre2.444)
Supplement: Supplementary file 1 — Supplementary Table 1: Search words and Boolean operators [file CRE2-7-1037-s001.docx]

Supplementary data

| Search Number | PICO - Population | Search Number | PICO - Population | |  |
| --- | --- | --- | --- | --- | --- |
| S1 | Grade V Implant* | S15 | Implant* alloy corros* | |  |
| S2 | Grade 5 Implant* | S16 | Ti-6Al-4V corros* | |  |
| S3 | Small diameter implant | S17 | Implant* alloy particle* | |  |
| S4 | Implant* alloy | S18 | Ti-6Al-4V particle* | |  |
| S5 | Ti-6Al-4V | S19 | Implant* alloy ion* | |  |
| S6 | Orthodontic implant* | S20 | Ti-6Al-4V ion* | |  |
| S7 | Mini implant* | S21 | Implant* alloy wear | |  |
| S8 | abutment alloy | S22 | Ti-6Al-4V wear | |  |
| S9 | Biomedical implant* | S23 | S15 OR S16 OR S17 OR S18 OR S19 OR S20 OR S21 OR S22 | |  |
| S10 | Joint replace* | Search Number | PICO - Outcome | |  |
| S11 | Arthroplasty | S29 | Fibroblast* | |  |
| S12 | MH Arthroplasty adverse effect | S30 | fibroblast* cytotoxic* | |  |
| S13 | MH titanium alloy, adverse effect | S31 | fibrobast* toxic* | |  |
| S14 | S1 OR S2 OR S3 OR S4 OR S5 OR S6 OR S7 OR S8 OR S9 OR S10 OR S11 OR S12 OR S13 | S32 | Fibroblast* apoptosis | |  |
|  |  | S33 | Fibroblast* prolifer* | |  |
| Search Number | PICO - Comparison | S34 | MeSH Fibroblast cytoxic | |  |
| S24 | Grade IV implant* |  | S29 OR S30 OR S31 OR S32 OR S33 OR S34 | |  |
| S25 | Grade 4 Implant* |  |  | |  |
| S26 | Implant | PICO | S14 AND S23 AND S28 AND S34 | |  |
| S27 | MeSH Prosthesis and Implant |  |  | |  |
| S28 | S24 OR S25 OR S26 OR S27 |  | |  | |

Supplement table 1. Search words and Boolean operators
